# A Cumulant-Based Method for Acquiring GNSS Signals

**DOI:** 10.3390/s24196234

**Published:** 2024-09-26

**Authors:** He-Sheng Wang, Hou-Yu Wang, Dah-Jing Jwo

**Affiliations:** Department of Communications, Navigation and Control Engineering, National Taiwan Ocean University, 2 Peining Rd., Keelung 202301, Taiwan; hswang@email.ntou.edu.tw (H.-S.W.); hywang.why@gmail.com (H.-Y.W.)

**Keywords:** GNSS, Galileo, BOC, higher-order cumulant

## Abstract

Global Navigation Satellite Systems (GNSS) provide positioning, velocity, and time services for civilian applications. A critical step in the positioning process is the acquisition of visible satellites in the sky. Modern GNSS systems, such as Galileo—developed and maintained by the European Union—utilize a new modulation technique known as Binary Offset Carrier (BOC). However, BOC signals introduce multiple side-peaks in their autocorrelation function, which can lead to significant errors during the acquisition process. In this paper, we propose a novel acquisition method based on higher-order cumulants that effectively eliminates these side-peaks. This method is capable of simultaneously acquiring both conventional ranging signals, such as GPS C/A code, and BOC-modulated signals. The effectiveness of the proposed method is demonstrated through the acquisition of simulated signals, with a comparison to traditional methods. Additionally, we apply the proposed method to real satellite signals to further validate its performance. Our results show that the proposed method successfully suppresses side-peaks, improves acquisition accuracy in weak signal environments, and demonstrates potential for indoor GNSS applications. The study concludes that while the method may increase computational load, its performance in challenging conditions makes it a promising approach for future GNSS receiver designs.

## 1. Introduction

As noted by Hofmann-Wellenhof, Lichtenegger, and Wasle [1], the Global Navigation Satellite System (GNSS) is a space-based radio positioning system that includes one or more satellite constellations, augmented as necessary to support the intended operation. GNSS provides 24-h, three-dimensional positioning, velocity, and time services to suitably equipped users anywhere on or near the surface of the Earth. In recent years, modern users have gained access to several additional GNSS options due to the development or modernization of systems like Russia’s GLObal NAvigation Satellite System (GLONASS) and China’s COMPASS constellation [2]. GLONASS, which is very similar to GPS, is owned and operated by the military and is designed to function with a full constellation of 24 satellites. Another GNSS system, the Galileo positioning system, is being jointly developed by the European Union (EU) and the European Space Agency (ESA).

To efficiently reuse RF bandwidth, Galileo employs a novel modulation technique called Binary Offset Carrier (BOC), which is intended to offer better service than other GNSS systems. However, this technique can introduce multiple side-peaks during the acquisition process. Recent studies [3,4] have addressed this issue. In a conventional GNSS receiver, the positioning process includes RF front-end processing, acquisition, tracking, and navigation data decoding. The acquisition process aims to identify signals from visible satellites and determine coarse values of Doppler frequencies and code phases of the ranging signals. Code Division Multiple Access (CDMA) is the primary technique used in GNSS signals, allowing users to distinguish each satellite signal obtained from the receiver antenna via different Coarse/Acquisition (C/A) codes, also known as satellite identification numbers or PRN numbers, during the acquisition process. Once the C/A code of a satellite is found, the signal can be tracked. The purpose of this paper is to introduce a novel acquisition method based on higher-order cumulants that efficiently suppresses side-peaks when acquiring Galileo signals.

A cumulant is a higher-order statistical expectation. Over the past few decades, higher-order statistics (HOS) have been applied across various fields, including sonar, radar, speech/image processing, geophysics, and biomedical signal processing [5,6,7]. Among these, significant research has focused on time-delay estimation [8,9,10]. Swami, Mendel, and Nikias [11] extensively discussed the application of HOS to time-delay estimation, particularly useful when the noise in the signal is non-Gaussian. The GNSS signal acquisition process is essentially a time-delay estimation problem. However, the signal model in Swami et al. is not suitable for GNSS signal processing due to the very low signal-to-noise ratio. To overcome this limitation, we propose a new method that allows the use of cumulants to acquire GNSS signals effectively.

GNSS signal acquisition involves a two-dimensional search process, requiring the exploration of all potential spreading code phases and Doppler frequencies. To enhance search efficiency, parallel acquisition techniques have been developed. These methods utilize fast Fourier transforms (FFT) to convert the signal from the time domain to the frequency domain, enabling rapid processing. Parallel acquisition methods can be classified into three main types:

(a) Parallel Frequency Search: This approach utilizes FFT to simultaneously explore all or a subset of Doppler frequency bins, allowing for an efficient search across the frequency domain [12,13].
(b) Parallel Code Search: In this method, FFTs are employed to compute code correlations, enabling the simultaneous examination of all code delay bins, thus expediting the search process within the code domain [14,15].
(c) Two-Dimensional Search: This technique applies FFTs to both the frequency and code domains, performing a simultaneous search across both dimensions, thereby enhancing acquisition speed and accuracy [16,17,18,19].

Acquisition is a critical step in the GNSS receiver’s positioning process, but issues such as false lock (e.g., code phase searching error) can occur. This can lead the GNSS receiver to incorrectly estimate the ranges between users and satellites, potentially resulting in positioning errors. Furthermore, the presence of multiple side-peaks in the BOC autocorrelation function makes Galileo satellite signal acquisition more challenging when using conventional methods. This study proposes a novel acquisition method in which we utilize the parallel FFT alongside the cumulant to suppress noise and reduce side-lobe effects during the acquisition of Galileo satellite signals.

The remainder of this paper is organized as follows: Section 2 provides a brief introduction to the Galileo system and BOC signals. Section 3 describes the proposed acquisition method in detail. Simulation and experimental results are presented in Section 4. Finally, conclusions are drawn in Section 5.

## 2. Galileo Overview

Today the main modulation technique used in the Galileo signal is Composite Binary Offset Carrier (CBOC) Modulation [14,20,21], which is composed of two different BOC signals. CBOC consists of a lower frequency BOC component (e.g., BOC(1,1)) and a higher frequency BOC component (e.g., BOC(6,1)). The description of CBOC is given as CBOC(m1, m2, r), where m1 and m2 are the two sub-carriers, respectively, and r is the power ratio of the first sub-carrier relative to the total signal power.

Taking the Galileo E1 signal, for example, the primary code is modulated by CBOC(6,1,1/11). In the following we describe and combine all elements necessary to generate the Galileo L1 OS signal.

The transmitted bandwidth is 40 × 1.023 MHz = 40.92 MHz. The chip length of the ranging code is

(1) $$T_{c,L1-B}=T_{c,L1-C}=1/1.023\;\mathrm{Mchip}/s=977.5\;\mathrm{ns}.$$

The corresponding ranging code rates are

$$R_{c,L1-A}=2.5\times 1/1.023\;\mathrm{Mchip}/s,$$

$$R_{c,L1-B}=1/T_{c,L1-B}=1.023\;\mathrm{Mchip}/s,$$

$$R_{c,L1-C}=1/T_{c,L1-C}=1.023\;\mathrm{Mchip}/s,$$

and the subcarrier rates are

$$R_{sc,L1-B,n}=R_{sc,L1-C,n}=1.023\;\mathrm{MHz}\times n.\;(\mathrm{for}\;\mathrm{CBOC}(6,\;1,\;1/11),\;n=1,\;6)$$

The signal components for channel B results from modulo-2 addition of the navigation data stream $d_{L1-B}$, the PRN code sequence $c_{L1-B}$, and the B subcarrier $sc_{L1-B}$. The final component is denoted as $e_{B}$. Similarly, the C channel results from the modulo-2 addition of the C channel PRN code sequence $c_{L1-C}$ with the C channel subcarrier $sc_{L1-C}$. The component is $e_{C}$. The signal components are as follows:

(2) $$e_{B}\left(t\right)=\sum_{-\infty }^{\infty }\left(c_{L1-B,\left(i\;mod\;4092\right)}d_{L1-B,\left(i\;mod\;4\right)}{rect}_{T_{c,L1-B}}\left(t-iT_{c,L1-B}\right)\times sign\;\left(\sin\left(2\pi R_{c,L1-B}t\right)\right)\right)$$

(3) $$e_{C}\left(t\right)=\sum_{-\infty }^{\infty }\left(c_{L1-C,\left(i\;mod\;4092\right)}d_{L1-C,\left(i\;mod\;4\right)}{rect}_{T_{c,L1-C}}\left(t-iT_{c,L1-C}\right)\times sign\;\left(\sin\left(2\pi R_{c,L1-C}t\right)\right)\right)$$

**Figure 1:**
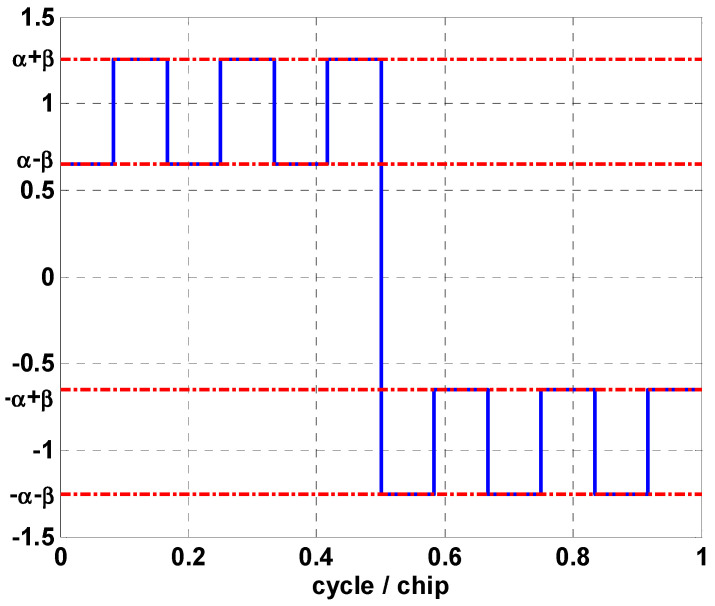
One period of the CBOC sub-carrier for E1 signal component.

**Figure 2:**
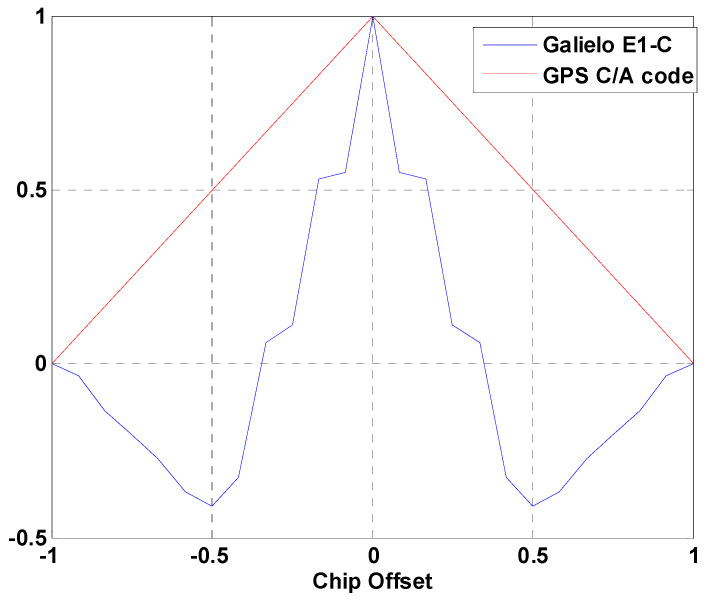
Comparison of autocorrelation between GPS C/A code and Galileo E1-C.

Here rect*_T_* ( ) is a rectangular function which is equal to 1 for 0 < *t* < *T* and equal to 0 elsewhere. The E1 channel signal can then be represented as follows:

(4) $$s(t)=\frac{1}{\sqrt{2}}(e_{B}(t)(\alpha sc_{L1-B,6}(t)+\beta sc_{L1-B,1}(t))-e_{C}(t)(\alpha sc_{L1-C,6}(t)-\beta sc_{L1-C,1}(t))).$$

with $sc_{X}(t)=\mathrm{sgn}(\sin(2\pi R_{sc,X}t))$. In this case, the parameters α and β are chosen to match the CBOC(6,1,1/11). That is, $\alpha =\sqrt{10/11}$ and $\beta =\sqrt{1/11}$. One period of the sub-carrier for the E1 signal component is shown in Figure 1. Figure 2 compares the primary code modulated by CBOC with the GPS C/A code. It is evident that the autocorrelation of the CBOC signal exhibits three peaks, which presents a significant challenge that many researchers are keen to address.

## 3. Cumulant-Based Acquisition Method

### 3.1. Definition of Cumulants

In this section, we will provide a brief overview of the definition of cumulants. Considering a one-dimensional, zero-mean random process x(n) with finite length, the first-order cumulant of this process is simply the mean:

(5) $$C_{1x}:=E\{x(n)\}$$

For a zero-mean stationary process, the second-, third-, and fourth-order cumulants are given by [22]:

(6) $$C_{2x}(\tau_{1}):=E\{x^{*}(n)x(n+\tau_{1})\}$$

(7) $$C_{3x}(\tau_{1},\tau_{2}):=E\{x^{*}(n)x(n+\tau_{1})x(n+\tau_{2})\}$$

(8) $$\begin{array}{c} C_{4x}(\tau_{1},\tau_{2},\tau_{3}):=E\{x^{*}(n)x(n+\tau_{1})x(n+\tau_{2})x^{*}(n+\tau_{3})\}\\ \;\;\;\;-C_{2x}(\tau_{1})C_{2x}(\tau_{2}-\tau_{3})-C_{2x}(\tau_{2})C_{2x}(\tau_{1}-\tau_{3})-M_{2x}^{*}(\tau_{3})M_{2x}(\tau_{1}-\tau_{2}) \end{array}$$

where E{•} denotes expectation, and $M_{2x}(\tau )=E\{x(n)x(n+\tau )\}=C_{2x}(\tau )$ for a real-valued process. The higher-order cumulants are invariant to a shift of mean. Therefore, one should replace x(n) with x(n)−E{x(n)} in the case of a nonzero mean process. As previously mentioned, first- and second-order cumulants correspond to the mean and variance, respectively, and are not applicable for time-delay estimation. For Gaussian noise, the fourth-order cumulants are approximately zero, making them unsuitable for GNSS signals. In this paper, we focus on using third-order cumulants for signal acquisition.

Here are some important properties of cumulants that are relevant to our study:

- If x(n) is symmetrically distributed, then $C_{3x}(\tau_{1},\tau_{2})=0$. If x(n) is Gaussian distributed, then $C_{4x}(\tau_{1},\tau_{2},\tau_{3})=0$.
- If λ is a constant, then $C_{kx}\{x(n)+\lambda \}=C_{kx}\{x(n)\}$ for k>2, where $C_{kx}\{•\}$ denotes *k*th-order cumulant.
- If y(n)=x(n)+w(n), where x(n) and w(n) are statistically independent random process, then $C_{ky}\{y(n)\}=C_{kx}\{x(n)\}+C_{kw}\{w(n)\}$, with similar relationships holding for cumulants of all orders.
- Assuming w(n) is Gaussian (colored or white) and independent of x(n), then for k>2, $C_{ky}\{y(n)\}=C_{kx}\{x(n)\}$. Hence, cumulants can recover the non-Gaussian signals even in the presence of colored Gaussian noise.

### 3.2. FFT-Cumulant Method for GNSS Signal Acquisition

As previously noted, the Gaussian nature of GNSS signals makes the direct application of the cumulant method largely ineffective. To address this challenge, we first apply an FFT to the signal and then compute its third-order cumulant in the frequency domain. To begin, we must define our signal model for the Galileo E1 signal. Let’s assume that the receiver measurement y(t) satisfies the following equation:

(9) $$y(t)=\sum_{k\in N}c^{k}(t-\tau^{k})c_{sc}(t)\cos(2\pi (f_{1}+f_{D}^{k})t+\theta^{k})+w(t),N\in [1,32]$$

where superscript *k* denotes the satellite PRN number, $c^{k}(t-\tau^{k})$ is the Galileo Primary code with different delay, and $\tau^{k}$; $c_{sc}(t)$ is the square wave sub-carrier used on BOC modulation; d(t) is the navigation data; $\cos(2\pi (f_{1}+f_{D}^{k})t+\theta^{k})$ is the carrier signal on L1 band with satellite-to-receiver Doppler shift $f_{D}^{k}$ and carrier phase $\theta^{k}$ with respect to the local reference oscillator; and w(t) is an unknown noise source. We shall now drop the superscript for simplicity, resulting in the following:

(10) $$y(t)=c(t-\tau )c_{sc}(t)\cos(2\pi (f_{1}+f_{D})t+\theta )+w(t)$$

The receiver’s RF front-end performs several critical functions on the signal received from the satellite, including mixing, frequency down-conversion, and low-pass filtering. These operations produce a baseband signal that is suitable for further processing, as shown below:

(11) $$y^{\prime }(t)=c(t-\tau )c_{sc}(t)\cos(2\pi (f_{IF}+f_{D})t+\theta )+w(t)$$

The method proposed in this study can be implemented using two different approaches, as described below:

Approach #1:

1. Carrier Signal Multiplication: The received signal is first multiplied by a locally generated carrier signal. This process generates two components: the in-phase (I) signal, by multiplying with the local carrier, and the quadrature (Q) signal, by multiplying with a phase-shifted version of the carrier. These two signals are then combined to form the complex input signal s(t)=I(t)+jQ(t).
2. PRN Sequence Multiplication: the complex signal is then multiplied by locally generated PRN (Pseudo-Random Noise) sequences.
3. Frequency-Domain Transformation: after multiplication with the PRN sequence, a Discrete Fourier Transform (DFT) is performed on the signal to convert it into its frequency-domain representation.
4. Cumulant Estimation: The third-order cumulant is estimated from the frequency-domain signal. The absolute value of the resulting output represents the correlation in the frequency-cumulant domain.

**Figure 3:**
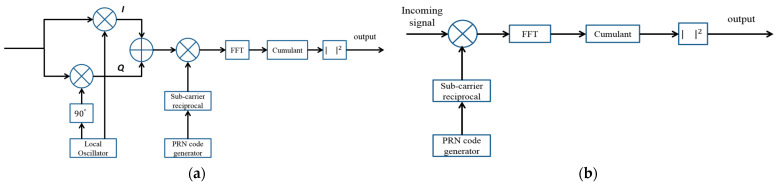
(**a**) Block diagram of the FFT-Cumulant method; (**b**) Block diagram of the Modified FFT-Cumulant method.

The above operation is illustrated in Figure 3a. In the second step, the signal is multiplied by a known PRN sequence with a code phase delay ranging from 0 to 4091. In Step 1, the Doppler shift and carrier phase are effectively removed, resulting in a signal that is ready for further processing:

(12) $$\tilde{y}\left(t\right)=c\left(t-\tau \right)c_{sc}(t)+w(t)$$

This yields a cleaned signal, free from Doppler effects and aligned with the locally generated PRN sequence, allowing for accurate subsequent analysis. Next, by multiplying the signal from Equation (12) by a local replica of the PRN code, which is modulated by the sub-carrier reciprocal term, we obtain

(13) $$\tilde{y}(t)c(t-\hat{\tau })c_{sc}^{-1}(t)=c(t-\tau )c_{sc}(t)c(t-\hat{\tau })c_{sc}^{-1}(t)+w(t)c(t-\hat{\tau })$$

Here, ‘^’ indicates estimation. If $\hat{\tau }$ is shifted to align with the real τ, then

(14) $$\tilde{y}(t)=c^{2}(t-\tau )+\hat{w}(t)$$

It is easy to see that the term $c^{2}(t-\tau )$ is a constant, where all elements are equal to one. When we take the Fourier transform in Step 4, it is well-known that the Fourier transform of a constant yields an impulse in the frequency domain. As a result, we obtain a sequence of data with one large value while all other values are equal to zero. Therefore, Equation (14) simplifies to

(15) $$FFT\{\tilde{y}(t)\}=FFT\{c{\left(t-\tau \right)}^{2}\}+FFT\{\hat{w}(t)\}$$

Then, from the cumulant property 1 (Cumulant additivity):

(16) $$C_{3}\{FFT\{\tilde{y}(t)\}\}=C_{3}\{FFT\{c^{2}(t-\tau )\}\}+C_{3}\{FFT\{\hat{w}(t)\}\}$$

In this context, *FFT*{•} denotes the fast Fourier transform operation. Since $\hat{w}(t)$ is a real signal, the second term $FFT\{\hat{w}(t)\}$ in the frequency domain is a symmetric function, which can be disregarded due to the properties of cumulants.

While this method offers strong noise suppression capabilities, it involves conducting two separate searches (for code phase and carrier), which leads to a significant computational burden. To enhance computational efficiency, we propose a second algorithm, described below.

Approach #2:

1. The signal from Equation (11) is multiplied by a locally generated PRN code modulated with the BOC subcarrier reciprocal.
2. A Fourier Transform is performed on the resulting signal.
3. The cumulant is estimated.

The implementation of this approach is straightforward and can be directly executed using the steps outlined above, following the block diagram shown in Figure 3b. Multiplying the signal in Equation (11) by the replica of the PRN sequence results in

(17) $$\tilde{y}\left(t\right)c\left(t-\hat{\tau }\right)c_{sc}^{-1}(t)=c\left(t-\tau \right)c_{sc}\left(t\right)c\left(t-\hat{\tau }\right)c_{sc}^{-1}(t)\cos(2\pi (f_{IF}+f_{D})t+\theta )+w(t)c(t-\hat{\tau })$$

If $\hat{\tau }$ matches with τ, the term $c(t-\tau )c_{sc}(t)c(t-\hat{\tau })c_{sc}^{-1}(t)$ becomes one, and (15) can be rewritten as

(18) $$\tilde{y}(t)=\cos(2\pi (f_{IF}+f_{D})t+\theta )+\hat{w}(t)$$

Equation (18) is simply a sinusoidal wave added with an unknown noise. Taking Fourier transform on (18), we obtain

(19) $$FFT\{\tilde{y}(t)\}=FFT\{\cos(2\pi (f_{IF}+f_{D})t+\theta )\}+FFT\{\hat{w}(t)\}$$

The first term on the right-hand side exhibits two peaks in the frequency domain. Consequently, after performing the cumulant estimation on Equation (19), we obtain a large value, which can be expressed as

(20) $$C_{3}\{FFT\{\tilde{y}(t)\}\}=C_{3}\{FFT\{\cos(2\pi (f_{IF}+f_{D})t+\theta )\}\}+C_{3}\{FFT\{\hat{w}(t)\}\}$$

As noted earlier, the second term on the right-hand side equals zero due to the properties of the cumulant. Compared to the previous approach, this modified FFT-cumulant method reduces the search space by focusing only on finding code phases, eliminating the need for a local oscillator. As shown in Figure 3b, Approach 2 simplifies the process by reducing several blocks compared to the previous method. This approach can also be easily extended to the GPS signal case by rewriting Equation (11) as follows:

$$y^{\prime }(t)=c(t-\tau )\cos(2\pi (f_{IF}+f_{D})t+\theta )+w(t)$$

In our study, we processed the data in batch mode, which presents challenges in providing precise quantitative measurements of the speed difference between the two approaches. Our typical dataset consisted of a few to several tens of seconds of sample data, approximately equivalent to a few hundred megabytes.

To estimate the computational time, we utilized Matlab’s tic-toc function. While this method doesn’t provide extremely precise measurements, it does offer a reasonable approximation of the processing duration. Our observations showed that Approach 1 typically required between 10 and 20 min of processing time for our dataset, while Approach 2 consistently completed in less than 10 min for the same dataset.

These results suggest that Approach 2 is approximately 1.5 to 2 times faster than Approach 1 in our implementation. However, it is important to note that the exact speed improvement can vary depending on factors such as the specific hardware used, the size of the dataset, and the particular signal characteristics being analyzed.

While these measurements provide a general indication of the relative efficiency of the two approaches, a more rigorous analysis in a real-time processing environment would be necessary to quantify the exact performance gains in practical applications. 

## 4. Simulation and Experimental Results

In this section, we will show the acquisition results for GPS and Galileo signals using both the traditional method and the proposed method. For the traditional method, we refer to the usual parallel code-phase search algorithm. See [14] for more details.

### 4.1. Simulated Signal Acquisition

**Figure 4:**
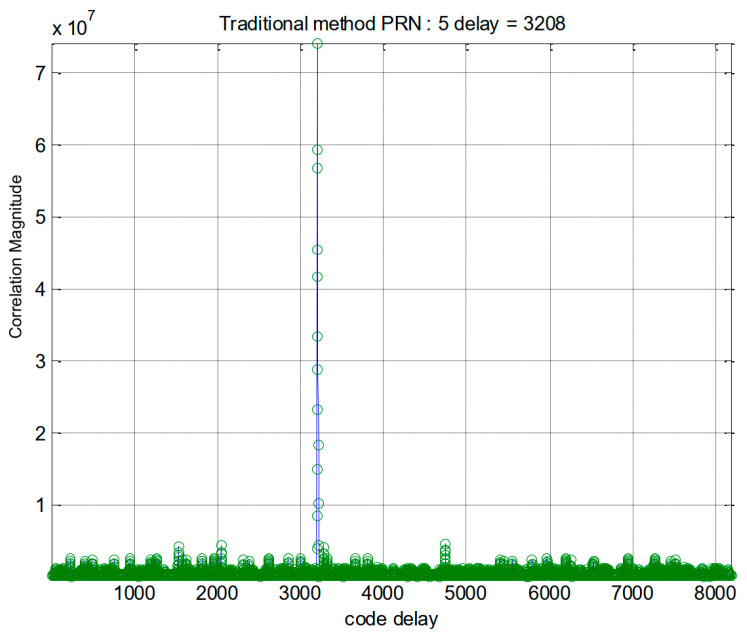
Acquisition results of the traditional method for GPS PRN 5.

**Figure 5:**
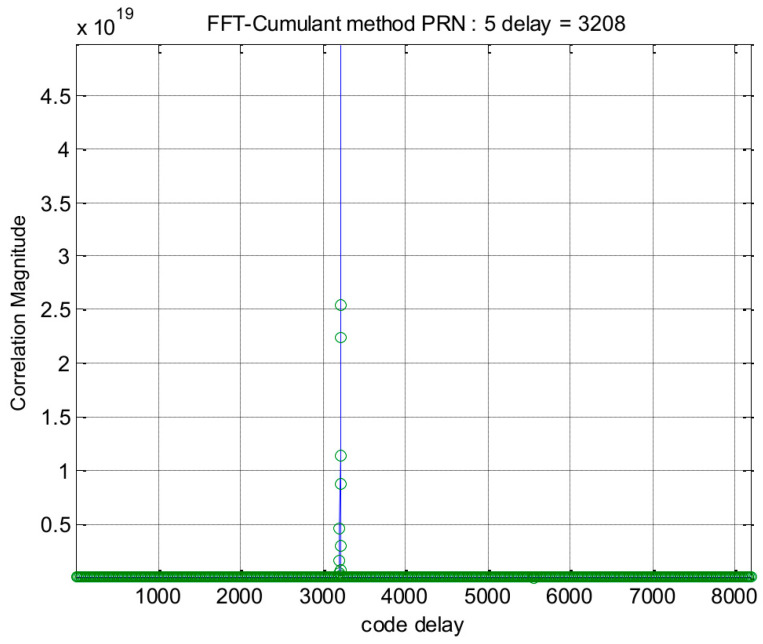
Acquisition results of the proposed method for GPS PRN 5.

In the first part of this section, we present some simulation results. The simulations were conducted on a standard desktop computer using MATLAB programs. For GPS signals, the results of the traditional method and the proposed method are shown in Figure 4 and Figure 5, respectively. At first glance, it might appear that the proposed method offers no clear advantages. However, it is important to note that our method exhibits a relatively higher processing gain. This suggests that our approach may have greater potential for applications involving weak signals. More details will be given at the end of this section.

**Figure 6:**
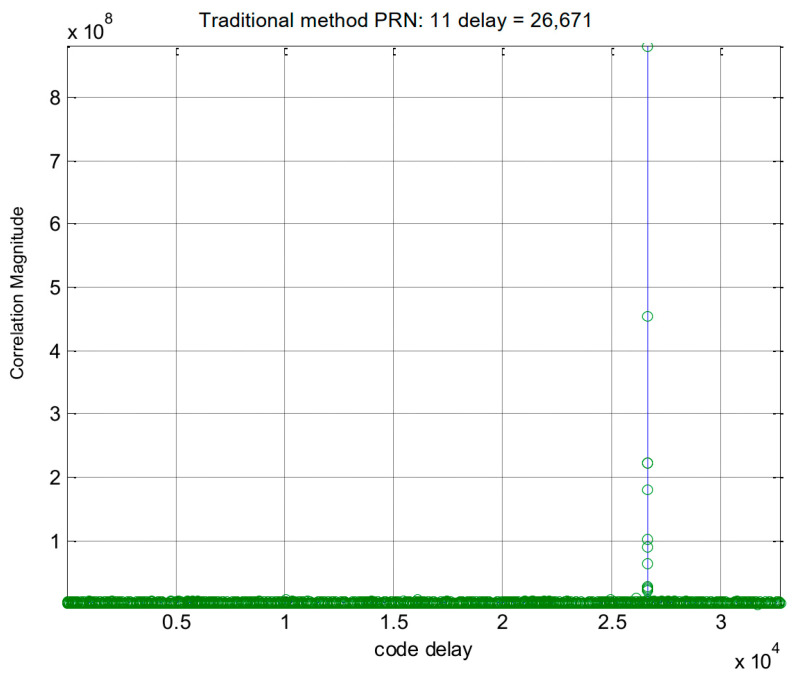
Acquisition results of the traditional method for Galileo PRN 11.

**Figure 7:**
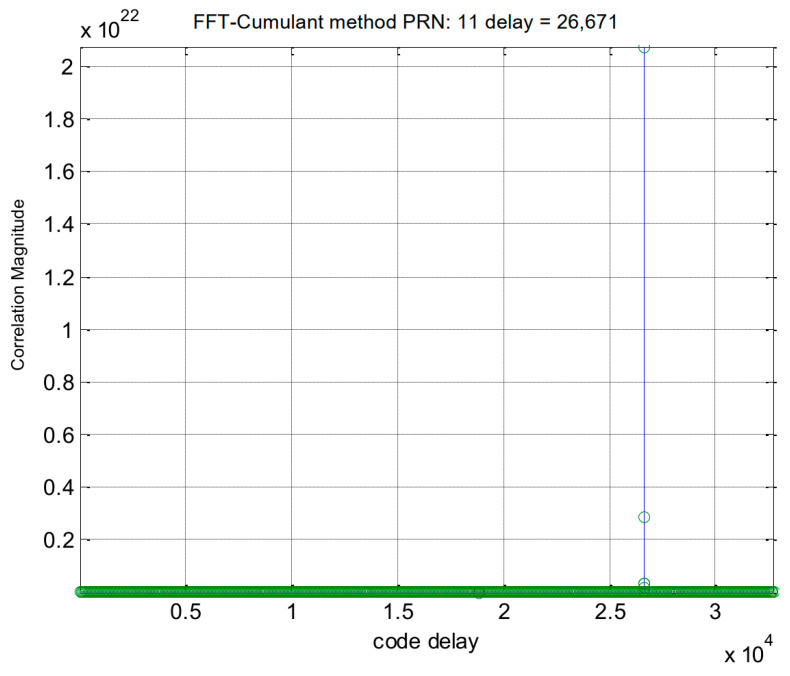
Acquisition results of the proposed method for Galileo PRN 11.

**Figure 8:**
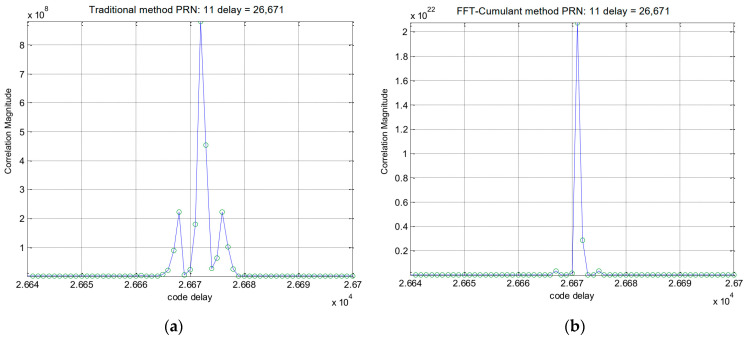
(**a**) Partial enlarged view of Figure 6. (**b**) Partial enlarged view of Figure 7.

The simulation procedure for the Galileo signal is similar to that used for the GPS signals. In this case, we selected the E1-C channel as our simulation signal (i.e., CBOC(6,1,1/11)). For Galileo PRN 11, the results obtained using the traditional method and the proposed method are displayed in Figure 6 and Figure 7, respectively. Figure 8 provides a zoomed-in view of the acquisition results. As shown in these figures, the proposed method demonstrates effective side-peak suppression when acquiring the Galileo signal, indicating its superior efficiency.

**Figure 9:**
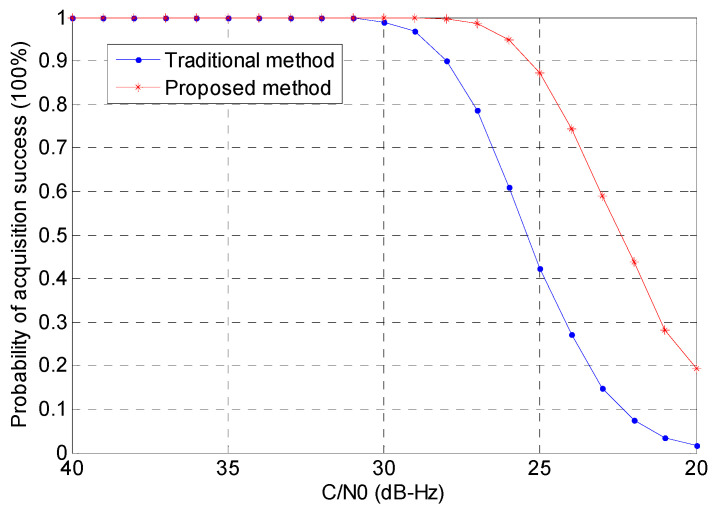
Probability of detection for the two methods (without multipath).

In the following section, we present several simulations to evaluate the performance of the different methods. The first case considers a multipath environment. In this scenario, the signal is assumed to follow a two-path channel model, as described in [23]:

(21) h(t)=δ(t)+αδ(t−β)

where α is the attenuation parameter of the second path, β is the time delay of the second path relative to the first path, and δ(t) is the Dirac-delta function. A Monte Carlo simulation is conducted, with α set to 0.6 and β randomly assigned at each iteration. Figure 9 shows the results of the probability of detection against SNR. In this simulation, acquisition is considered successful if the following conditions are met:

1. The ratio between the main peak and the second peak is greater than 2.5.
2. The position of the main peak corresponds to the correct code phase delay.

**Figure 10:**
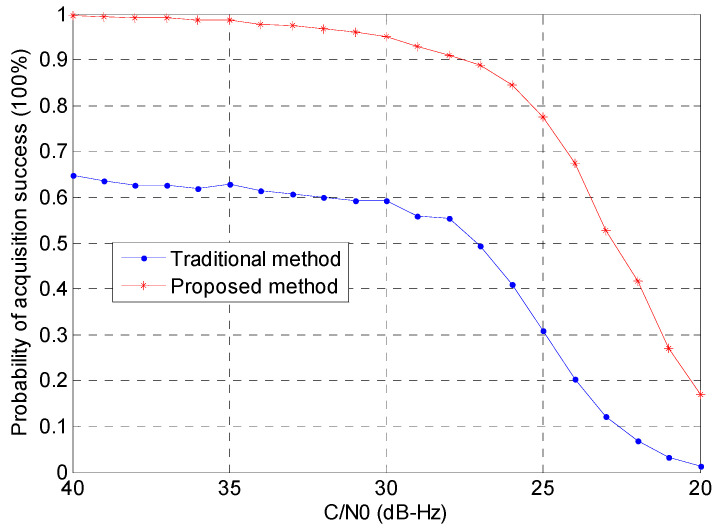
Probability of detection for the two methods (with multipath).

Figure 10 presents the results of the probability of detection in a multipath environment. In both scenarios, it is evident that the proposed method outperforms the traditional approach. Notably, the proposed method maintains a detection rate of over 50% when the signal level is around 23 dB-Hz. This signal level is significantly below what is typically required for off-the-shelf receivers, indicating that our method may have considerable potential for indoor applications where signal strength is often weaker.

In summary, as shown in Figure 8a,b, our proposed method effectively eliminates the side-peaks that are present in the traditional method’s acquisition results. This suppression of side-peaks significantly reduces the risk of false acquisition. Referring to Figure 9 and Figure 10, we can quantify the improvement in detection probability as follows:

− In a non-multipath environment (Figure 9), at a signal level of 25 dB-Hz, the proposed method achieves a detection probability of approximately 90%, compared to about 70% for the traditional method. This represents a 28.6% improvement in detection probability.
− In a multipath environment (Figure 10), the improvement is even more significant. At 25 dB-Hz, the proposed method maintains a detection probability of about 80%, while the traditional method drops to approximately 50%. This represents a 60% improvement in detection probability under challenging multipath conditions.

### 4.2. Real GNSS Signal Acquisition Results 

In the next part of this section, we evaluate the performance of our method using real signal data collected via a GNSS receiver. The device used to capture the GNSS satellite signals is a SiGe GN3S Sampler with version 2 firmware. The specific parameters of the data captured from this GNSS module are as follows:

- Sampling frequency: 8.1838 MHz
- Intermediate frequency: 38.400 KHz
- Data format: 2-bit I/Q samples (1-bit I & 1-bit Q) in binary format (sI0, sQ0, sI1, sQ1, sI2, sQ2…)

**Figure 11:**
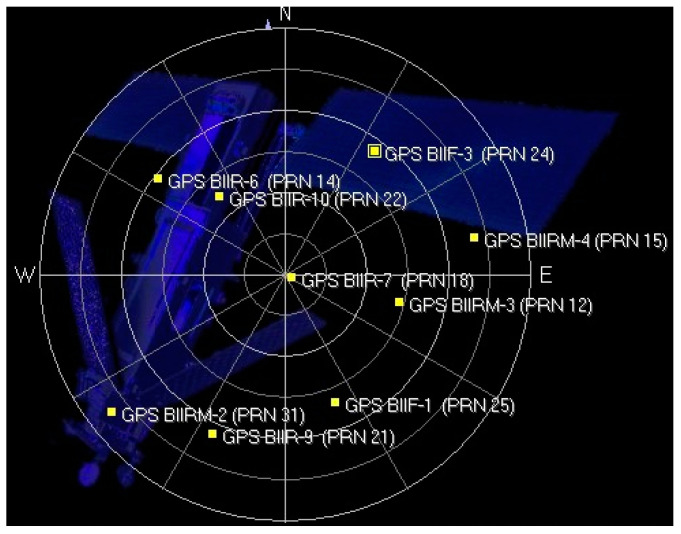
Sky plot showing the positions of visible GPS satellites during the experiment. Each numbered point represents a satellite, with its position on the plot indicating its azimuth and elevation relative to the receiver’s location. The blue structures in the background represent the local environment and are not relevant to the satellite data. The center of the plot represents the zenith, while the edge represents the horizon.

Figure 11 shows the sky plot of GPS satellites during the experiment. The experiment was conducted at approximately 12:40 p.m. local time. The elevations of each satellite observed during the experiment are listed in Table 1.

**Figure 12:**
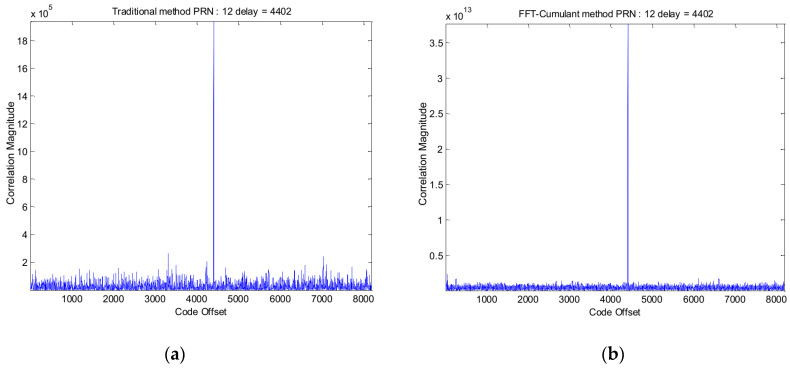
(**a**) GPS PRN 12 signal acquisition results using the traditional method; (**b**) GPS PRN 12 signal acquisition results using the proposed method.

Table 2 presents the satellite signal parameters (such as code phase and Doppler shift) acquired using the traditional method and the corresponding parameters obtained using the proposed method. Note that this is only for a matter of comparison, showing that both methods acquired the same code offset and Doppler shift; also note that PRN21 is not acquired using the traditional method. Figure 12a illustrates the acquisition results for PRN 12 using the traditional method, and Figure 12b shows the acquisition results for PRN 12 using the proposed method.

### 4.3. Real Galileo Signal Acquisition Results

**Figure 13:**
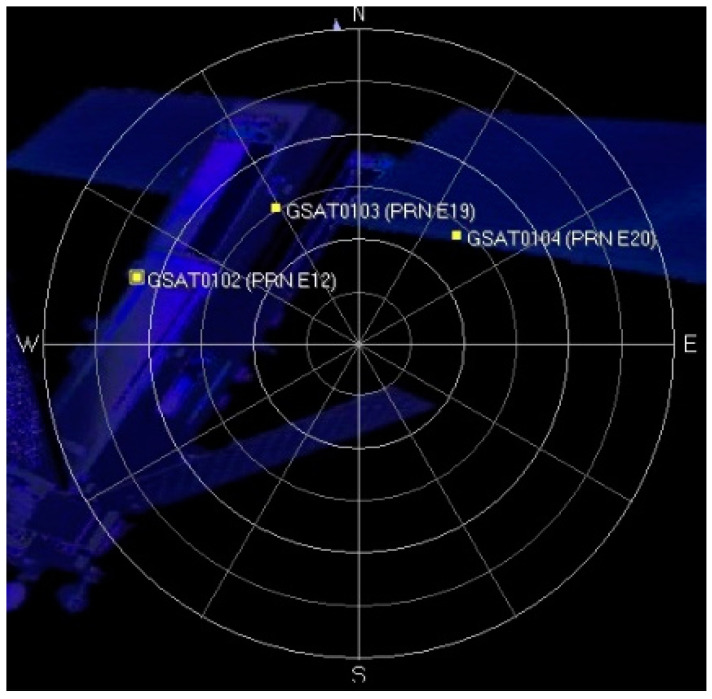
Sky plot showing the positions of visible Galileo satellites during the experiment. Each numbered point represents a satellite, with its position on the plot indicating its azimuth and elevation relative to the receiver’s location. The blue structures in the background represent the local environment and are not relevant to the satellite data. The center of the plot represents the zenith, while the edge represents the horizon.

To improve the reception of Galileo satellite signals, we utilized the SiGe GN3S Sampler version 3, an upgraded version of this USB dongle that features a wideband receiving mode. This new version allows users to select different modes (narrowband for GPS or wideband for Galileo) to capture various satellite signals. The specific parameters for these different modes are listed in Table 3. For this experiment, we selected Mode 6 to capture the signal. Figure 13 shows the sky plot of Galileo satellites observed from the university campus at 5:05 PM.

**Figure 14:**
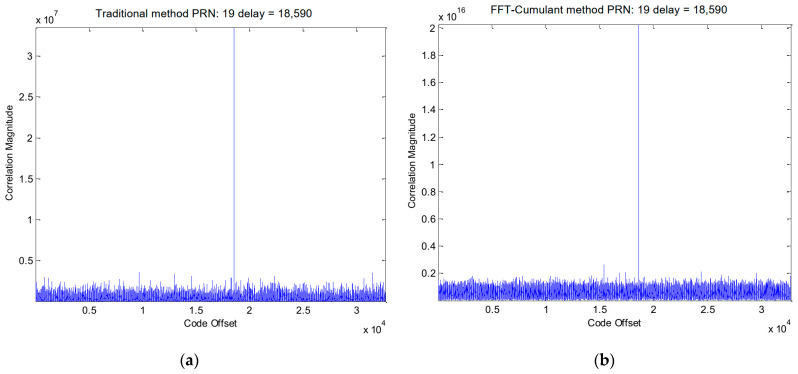
(**a**) GALILEO PRN 19 signal acquisition results using the traditional method; (**b**) GALILEO PRN 19 signal acquisition results using the proposed method.

Table 4 provides a summary of the acquisition results using the traditional method and the proposed method, respectively. Again, the two methods acquire the same code phase and Doppler shift. Figure 14a illustrates the acquisition results for PRN 12 using the traditional method, while Figure 14b depicts the acquisition results for PRN 12 using the proposed method.

**Figure 15:**
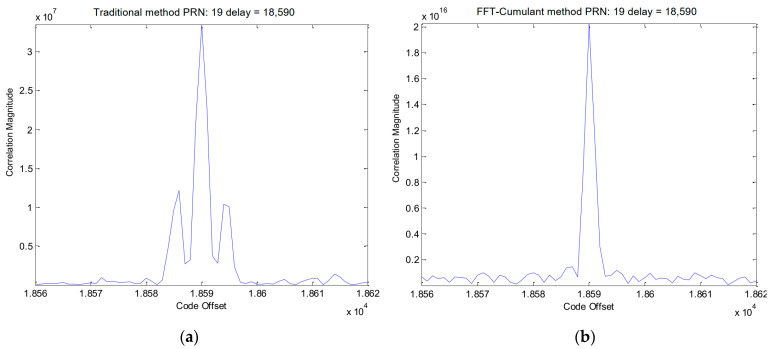
(**a**) Partial enlarged view of Figure 14a; (**b**) Partial enlarged view of Figure 14b.

Figure 15a,b presents enlarged local plots of the acquisition results for the Galileo PRN 19 E1-C channel signal using the traditional method and the proposed method, respectively. As observed in the figures, the traditional method exhibits two side-peaks near the correct peak position. In contrast, the proposed method effectively eliminates these side-peaks, resulting in a cleaner acquisition result.

### 4.4. Indoor GPS Signal Acquisition Results

**Figure 16:**
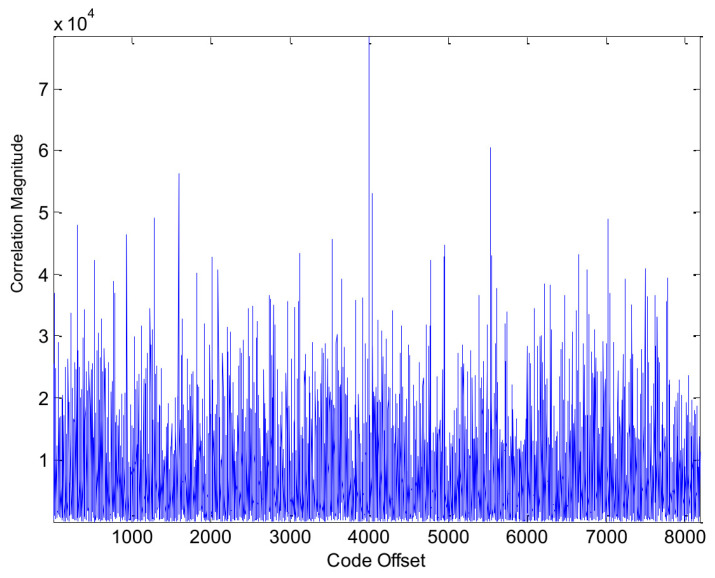
Acquisition results of GPS PRN5 using the traditional method in indoor conditions.

**Figure 17:**
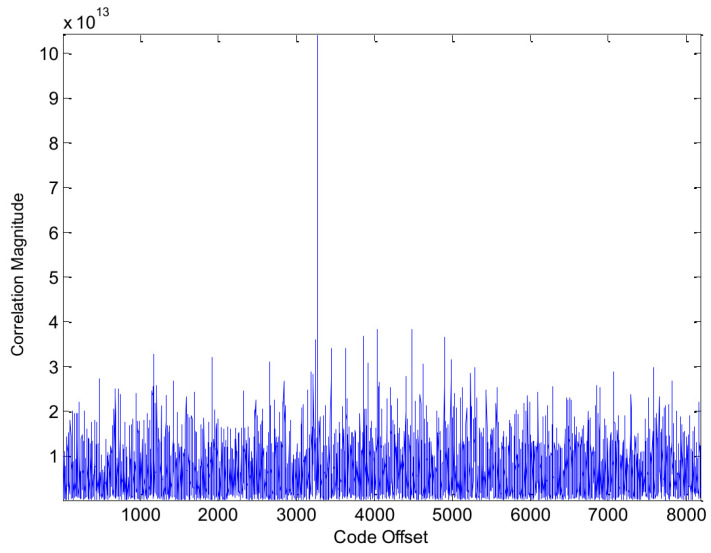
Acquisition results of GPS PRN5 using the proposed method in indoor conditions.

As a final experiment in this paper, we assess the capability of the proposed algorithm in acquiring weak signals. For this test, the receiver was placed inside a building, within one meter of a window on the local campus. Figure 16 and Figure 17 display the acquisition results using the traditional method and the proposed method, respectively. As seen in Figure 16, the traditional method struggles to correctly identify the code phase delay, with the peak occurring at the wrong code-phase position. In contrast, Figure 17 shows that the proposed method successfully identifies the correct code-phase position, with a clear and distinct peak.

## 5. Discussion

The results of this study demonstrate the efficacy of the proposed method for GNSS signal acquisition, particularly in challenging environments, such as those involving weak signals or multipath interference. Compared to traditional methods, our approach shows improvements in both the suppression of side-peaks and the accuracy of code-phase detection.

The problem of side-peaks in the acquisition of BOC-modulated signals, as noted in previous studies [3,4], has been a persistent challenge due to the complex autocorrelation function of these signals. Traditional methods often fail to completely eliminate these side-peaks, which can lead to false locks and positioning errors. Our results indicate that the proposed cumulant-based method effectively addresses this issue, eliminating side-peaks and improving the robustness of the signal acquisition.

Moreover, the higher processing gain observed in our method suggests that it may be particularly valuable for applications involving weak signals, where traditional approaches struggle to maintain reliable acquisition. This finding aligns with the hypothesis that leveraging higher-order statistical properties, such as cumulants, can enhance signal detection in low-SNR environments. The ability of our method to detect signals at levels as low as 23 dB-Hz—below the typical thresholds for standard receivers—further supports this claim and highlights its potential for indoor and urban canyon applications, where signal strength is often compromised.

While the results are promising, there are several avenues for future research. First, further investigation is needed to optimize the algorithm’s performance under various noise conditions and to ensure its scalability for real-time processing in commercial GNSS receivers. Additionally, exploring the integration of this method with advanced signal processing techniques, such as machine learning, could provide further enhancements in acquisition speed and accuracy.

While our study demonstrates promising results for GPS signal acquisition in indoor environments, we acknowledge that our current work does not include equivalent results for Galileo signals in similar conditions. The acquisition of Galileo signals indoors presents unique challenges due to its signal structure and complex multipath effects. Our preliminary experiments in this area have not yet yielded satisfactory results, highlighting the need for further research. We identify this as a crucial area for future investigation, with the potential to significantly advance indoor GNSS positioning techniques. Future work will focus on optimizing our proposed method specifically for indoor Galileo signal acquisition, aiming to extend the benefits of our approach to this important use case.

Finally, while our study focused on GNSS signals, the principles underlying the proposed method may have broader applications in other fields where time-delay estimation is critical, such as radar, sonar, and telecommunications. Future research could explore these potential cross-disciplinary applications, thereby extending the impact of this work beyond satellite navigation.

## 6. Conclusions

In this paper, we proposed an acquisition method for GNSS signal processing, demonstrating its ability to efficiently eliminate side-peaks, particularly when applied to real Galileo signals. The method also exhibited strong side-peak cancellation performance when acquiring GNSS signals. We presented two different approaches for implementing the proposed method, and while our focus was on GPS and Galileo signals, we believe this method can be extended to other GNSS systems, such as BeiDou or GLONASS.

The proposed method relies on cumulants and Fourier transforms, both of which are computationally intensive. Consequently, this approach may not improve the time-to-first-fix (TTFF); in fact, the TTFF may increase due to the added computational load, which is the primary drawback of our method. However, as the simulation results illustrate, the proposed method achieves a satisfactory detection rate even for signals with very low carrier-to-noise density ratios (C/N0). Additionally, a simple indoor experiment demonstrated that our method is capable of acquiring signals inside a building, suggesting its potential for superior performance in weak signal environments and indoor applications.

Future research should focus on an in-depth analysis of this method, particularly in terms of optimizing computational efficiency and exploring its applicability across different GNSS systems and challenging environments. We also recognize the need to investigate methods to reduce the computational burden without compromising the method’s robustness, which remains a crucial area for future work.

## Figures and Tables

**Table 1:** GPS satellites elevation.

PRN Number	Elevation
12	46.6
14	31.9
15	19.3
18	87.2
21	26.3
22	52.8
24	34.1
25	39.8
31	9.1

**Table 2:** Acquisition results of GPS signal for the traditional and proposed methods (PRN21 is not detected using the traditional method).

PRN Number	Traditional Method		Proposed Method	
	Code Offset	Doppler [Hz]	Code Offset	Doppler [Hz]
12	4402	2723	4402	2723
18	219	1974	219	1974
21	–	–	3836	−1039
22	5235	3816	5235	3816
24	2925	−1382	2925	−1382
25	2152	4346	2152	4346

**Table 3:** SiGe GN3S Sampler v3 specification parameters.

Mode Number	Frequency Band	Sampling Frequency	Intermediate Frequency	Data Format
1	Narrow	16.368 MHz	4.092 MHz	2 bit real
2	Narrow	8.184 MHz	0 MHz	4 bit I/Q
3	Narrow	5.456 MHz	1.364 MHz	2 bit real
4	Narrow	4.092 MHz	0 MHz	4bit I/Q
5	Wide	16.368 MHz	4.092 MHz	2 bit real
6	Wide	8.184 MHz	0 MHz	4 bit I/Q
7	Wide	5.456 MHz	1.364 MHz	2 bit real
8	Wide	4.092 MHz	0 MHz	4 bit I/Q

**Table 4:** Acquisition results of the Galileo signal using the traditional and proposed methods.

PRN Number	Traditional Method		Proposed Method	
	Code Offset	Doppler [Hz]	Code Offset	Doppler [Hz]
12	26,618	503	26,618	503
19	18,590	1145	18,590	1145
20	2198	33	2198	33

## Data Availability

The data that support the findings of this study are available upon reasonable request from the authors.
